# *Clock* gene polymorphism and scheduling of migration: a geolocator study of the barn swallow *Hirundo rustica*

**DOI:** 10.1038/srep12443

**Published:** 2015-07-22

**Authors:** Gaia Bazzi, Roberto Ambrosini, Manuela Caprioli, Alessandra Costanzo, Felix Liechti, Emanuele Gatti, Luca Gianfranceschi, Stefano Podofillini, Andrea Romano, Maria Romano, Chiara Scandolara, Nicola Saino, Diego Rubolini

**Affiliations:** 1 Dipartimento di Bioscienze, Università degli Studi di Milano, via Celoria 26, I-20133 Milano, Italy; 2 Dipartimento di Scienze dell’Ambiente e del Territorio e di Scienze della Terra (DISAT), Università di Milano-Bicocca, Piazza della Scienza 1, I-20126 Milano, Italy; 3 Swiss Ornithological Institute, Seerose 1, CH-6204 Sempach, Switzerland

## Abstract

Circannual rhythms often rely on endogenous seasonal photoperiodic timers involving ‘clock’ genes, and *Clock* gene polymorphism has been associated to variation in phenology in some bird species. In the long-distance migratory barn swallow *Hirundo rustica*, individuals bearing the rare *Clock* allele with the largest number of C-terminal polyglutamine repeats found in this species (Q_8_) show a delayed reproduction and moult later. We explored the association between *Clock* polymorphism and migration scheduling, as gauged by light-level geolocators, in two barn swallow populations (Switzerland; Po Plain, Italy). Genetic polymorphism was low: 91% of the 64 individuals tracked year-round were Q_7_/Q_7_ homozygotes. We compared the phenology of the rare genotypes with the phenotypic distribution of Q_7_/Q_7_ homozygotes within each population. In Switzerland, compared to Q_7_/Q_7_, two Q_6_/Q_7_ males departed earlier from the wintering grounds and arrived earlier to their colony in spring, while a single Q_7_/Q_8_ female was delayed for both phenophases. On the other hand, in the Po Plain, three Q_6_/Q_7_ individuals had a similar phenology compared to Q_7_/Q_7_. The Swiss data are suggestive for a role of genetic polymorphism at a candidate phenological gene in shaping migration traits, and support the idea that *Clock* polymorphism underlies phenological variation in birds.

The timing of seasonal activities has major fitness consequences and is subjected to intense natural selection^1^,^2^. In species experiencing seasonal peaks of resource availability, typically occurring at medium-high latitudes, proper matching of life-cycle events to such resource pulses is fundamental for achieving a high fitness. The timing of seasonal activities is often set by genetically controlled endogenous circadian and circannual rhythms^3^, which are synchronized with ecological conditions through external drivers^4^, among which photoperiod plays a pivotal role^5^. This seasonal photoperiodic timer involves ‘circadian clock’ genes, which are responsible for the onset and setting of circadian and circannual rhythmicity^6^,^7^,^8^. The circadian clock relies on an auto-regulated negative feedback loop, the ‘core circadian oscillator’ (CCO)^9^,^10^. In birds and mammals, the CLOCK transcription factor, encoded by the *Circadian Locomotor Output Cycles Kaput* (*Clock*) gene, plays a central role within the CCO, both by acting as a transcriptional activator of the CCO itself and by activating the transcription of several output genes^11^,^12^. The C-terminal domain of the *Clock* gene contains a series of polyglutamine residues (Poly-Q) which may vary in length among species as well as among individuals within populations^13^,^14^,^15^,^16^,^17^,^18^.

Latitudinal increase in the number of Poly-Q repeats across populations has been suggested to reflect adaptation to local regimes of circannual photoperiodic variation^14^,^16^,^19^. Similarly, Poly-Q polymorphism may control variation in photoperiodic responses among individuals from the same population, causing variation in phenology at the individual level^15^,^17^,^20^,^21^. Though the evidence for an association between *Clock* polymorphism and timing of seasonal events at the population level is scant, two recent studies conducted on a long-distance migratory bird, the barn swallow *Hirundo rustica*, have shown that a very rare *Clock* genotype, the heterozygous Q_7_/Q_8_ (c.a. 1.5% of the population), which contains the largest Poly-Q stretch observed in this species (Q_8_), has a delayed timing compared to the most frequent (94%) Q_7_/Q_7_ genotype^17^,^20^. These findings are consistent with the idea that larger Poly-Q stretches are associated with a delayed phenology^15^,^17^,^20^,^21^, and suggest that the Q_7_/Q_8_ genotype shows a delayed annual routine. Specifically, the first study, carried out on an Italian barn swallow population, showed that the onset of laying by Q_7_/Q_8_ females was delayed by 13 days relative to Q_7_/Q_7_ counterparts, corresponding to ca. 0.7 s.d. later than the mean of Q_7_/Q_7_ females^17^. In the second study, carried out on moulting barn swallows in the sub-Saharan wintering areas, wing feather moult of three Q_7_/Q_8_ individuals was found to be significantly delayed, by ca. 8/9 days, than Q_7_/Q_7_ birds^20^. In both studies, the phenology of the other rare genotype Q_6_/Q_7_ (4.8%)^17^, with a smaller Poly-Q stretch, did not differ from that of the common genotype Q_7_/Q_7_^17^,^20^.

To date, the analysis of individual variation in scheduling of migration events across the entire annual cycle in small-sized birds has been hampered by the technical difficulties of tracking large numbers of individuals from the same population. Here we exploit data retrieved from 64 individual barn swallows tracked along the entire annual migration cycle using miniaturized light-level geolocators^22^ to explore the association between *Clock* genotype polymorphism and scheduling of migration events in two barn swallow populations breeding in Southern Switzerland (Magadino) and Northern Italy (Po Plain). We expected that rare individuals bearing the heterozygous Q_7_/Q_8_ genotype should show a delayed scheduling of migration and wintering events compared to the phenotypic distribution of phenological traits of Q_7_/Q_7_ birds, complementing previous evidence concerning delayed timing of breeding and moult.

## Results and Discussion

Most (90.6%) of the 64 individuals tracked for an entire annual cycle (Switzerland, n = 26; Po Plain, n = 38) were homozygous Q_7_/Q_7_, as expected. In the Swiss population, we detected two Q_6_/Q_7_ males and a single Q_7_/Q_8_ female, while in the Po Plain we detected three Q_6_/Q_7_ individuals (two males, one female) and no Q_7_/Q_8_ birds.

**Figure 1:**
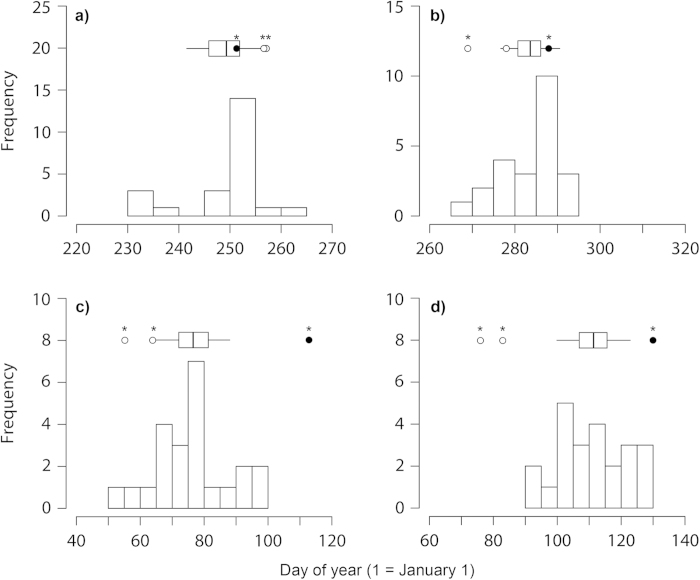
Frequency distribution of phenotypic values of the Q_7_/Q_7_ individuals and phenology of the Q_6_/Q_7_ and Q_7_/Q_8_ individuals in the Swiss population. (**a**) Departure from the breeding colony; (**b**) arrival to the wintering area; (**c**) departure from the wintering area; (**d**) arrival to the breeding colony. The horizontal boxes above histograms show the mean and 95% non-parametric bootstrap confidence interval (CI) of the Q_7_/Q_7_ phenotypic distribution, while the horizontal lines show the standard deviation. Dots show the phenotypic values of the rare genotypes (open dot = Q_6_/Q_7_; filled dot = Q_7_/Q_8_). Histograms and dots refer to actual data, while asterisks above dots denote that the Q_6_/Q_7_ or Q_7_/Q_8_ values were outside the 95% CI of a given phenological trait for centred values (removing any between-year and between-sex variation in phenology; see Supplementary Table S2 and Methods for details).

The migration and wintering phenology of Q_7_/Q_7_ birds showed a broad overlap between the two study populations, with the single exception of spring arrival date to the breeding colony, that was ca. 10 days later among Swiss birds compared to Po Plain ones (Fig. 1; Supplementary Table S1, Fig. S1; see also Liechti *et al*.^22^).

In the Swiss population, the single Q_7_/Q_8_ female showed a consistently delayed phenology and was outside the 95% confidence interval (CI) compared to the phenotypic distribution of Q_7_/Q_7_ for all traits (Fig. 1), both when actual or within-group centered values (thus removing any between-year and between-sex variation in phenology) were considered, the single exception being departure date from the breeding colony for actual values (Fig. 1a; Supplementary Table S2). On the other hand, the two Q_6_/Q_7_ males were consistently delayed and outside the 95% CI compared to the phenotypic distribution of Q_7_/Q_7_ individuals for departure from the breeding colony, but were consistently early and outside the 95% CI compared to Q_7_/Q_7_ for traits related to pre-breeding migration (Fig. 1c,d; Supplementary Table S2). They were instead not consistently advanced for arrival date to wintering area (Fig 1b; Supplementary Table S2). Results were similar if the analyses were separated by sex (Supplementary Table S3) and if we accounted for the effect of individual variation in wintering latitude and longitude (stationary wintering positions, SWP; see Methods) on arrival date to the breeding colony (Supplementary Table S2).

In the Po Plain, the lack of Q_7_/Q_8_ individuals allowed only a partial evaluation of the association between *Clock* polymorphism and phenology. Although being somewhat delayed in some cases (Supplementary Fig. S1, Table S2), the scheduling of migration and wintering events of the three Q_6_/Q_7_ was not consistently different from the phenotypic distribution of Q_7_/Q_7_ birds, with the exception of colony departure, that was delayed in all three individuals compared to the 95% CI of Q_7_/Q_7_ birds, similarly to the Swiss population (Supplementary Fig. S1, Table S2).

It may be worth noting that the two Swiss Q_6_/Q_7_ males and the Q_7_/Q_8_ female were also outside the CIs (advanced and delayed, respectively) of the Q_7_/Q_7_ Po Plain birds for departure from the wintering area and arrival to the breeding colony (cf. Fig. 1 and Supplementary Fig. S1; Table S2).

**Figure 2:**
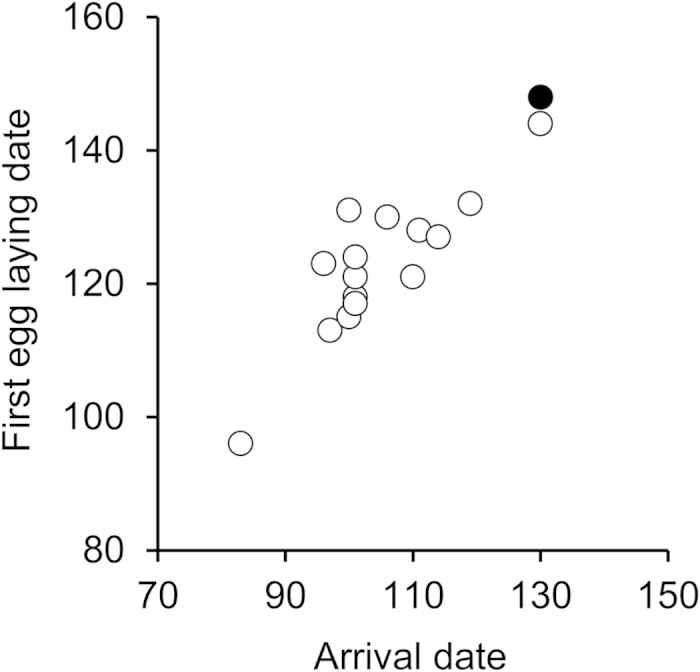
Arrival date predicts onset of reproduction among female barn swallows equipped with geolocators (day 1 = January 1^st^). The fitted regression equation is: first egg laying date = 0.88 (0.12 s.e.m.) × arrival date + 30.48 (12.51 s.e.m.) (R^2^ = 0.80). The filled dot denotes the single Q_7_/Q_8_ female from the Swiss population.

For the Swiss Q_7_/Q_8_ female, delayed arrival could lead to a delayed reproduction^17^. This female started egg laying on May 28^th^, 2012 (day 148), but unfortunately we have no other breeding data from geolocator females in the same year (nor from the same female in the previous year) for comparison. However, data from Q_7_/Q_7_ geolocator females from the previous year (spring 2011) revealed a mean first egg laying date that was ca. 20 days earlier (day 126, CI 120 to 136, n = 5) than the Q_7_/Q_8_ female. We are confident that the delayed laying of the Q_7_/Q_8_ female compared to Q_7_/Q_7_ females in the previous year was robust to between-year differences in egg laying dates: in fact, the Q_7_/Q_8_ female was a delayed phenodeviant for arrival date (Table 1; Fig. 1), and over the entire sample of geolocator females (both Swiss and Po Plain individuals) there was a strict association between arrival date and breeding date (*r* = 0.90, n = 16; Z*r* = 1.47, CI 0.93 to 2.01; Fig. 2). On the other hand, the two Swiss Q_6_/Q_7_ males did not have a clearly advanced breeding date compared to Q_7_/Q_7_ birds (details not shown for brevity). Indeed, the correlation between breeding date (start of egg laying by the social mate) and arrival date in geolocator males was weaker than in females (*r* = 0.31, n = 37; Z*r* = 0.32, CI −0.02 to 0.66), suggesting that timing of reproduction is mainly under female control^17^.

These findings, based on the first individual year-round tracking data for the barn swallow, complement previous evidence that the rare Q_7_/Q_8_ barn swallow genotype has a delayed timing^17^,^20^. Importantly, this evidence originates from the analysis of a further independent dataset, as no individuals from the Swiss population had been included in previous studies^17^,^20^. The delay of the Swiss Q_7_/Q_8_ individual was most pronounced for phenological variables related to timing of spring migration (departure from the wintering area and arrival date to the breeding colony) (Fig. 1).

Intriguingly, the two Swiss Q_6_/Q_7_ males showed an apparently advanced timing of spring migration compared to Q_7_/Q_7_ birds in the same population. In previous studies of the barn swallow, no statistically significant differences in timing emerged between Q_6_/Q_7_ and Q_7_/Q_7_ birds^17^,^20^. The Po Plain data show instead an inconsistent tendency towards a delayed pre-breeding migration phenology of the three Q_6_/Q_7_ birds compared to Q_7_/Q_7_ ones. Albeit not conclusively due to the small sample size of the ‘rare’ genotypes, the picture emerging from this and our earlier work may suggest that ‘clock’ genes differently affect phenology in different barn swallow populations, as previously documented by an analysis of the songbird genus *Junco*^18^. The most likely explanation for such among-population variation in ‘clock’ gene-phenology associations has been proposed to be variation in genetic background among different populations of the same species, with possibly other genes related to phenology variably interacting with *Clock* in different geographical populations^18^.

The proximate mechanisms leading to an association between length of Poly-Q stretches and phenology remain unknown, but may involve a different response to photoperiod or to other astronomical cues while in Africa. Despite photoperiodic changes around the Equator are minor, birds are able to detect them and tune the timing of their seasonal activities (including timing of moult) accordingly^23^. Moreover, it has recently been suggested that tropical birds can use variation in time of sunrise and sunset to adjust the timing of life-history events along the annual cycle^24^. Our findings suggest that the previously documented reproductive delay in Q_7_/Q_8_ females was likely to be mainly a consequence of carry-over effects of events occurring during wintering (such as moult) and at the onset of spring migration. Elucidating whether the *Clock*-phenology association causally stems from variable photoperiodic responses while in the African wintering grounds, triggering variable onset of migration activities with carry-over effects on timing of reproduction, would be a challenge for future research.

The fact that the association between *Clock* genotype and timing of autumn migration was somewhat less clear may be due to the difficulties in identifying the onset of autumn migration. Geolocator data allow only an estimate of the timing of departure from the breeding colony, which may not be indicative of variation in onset of post-breeding migration. In fact, the timing of departure from the breeding colony may be heavily affected by involvement in reproductive activities (e.g. nest failure, laying of multiple clutches and extent of post-fledging parental care) besides timing of reproduction. In addition, departures are very concentrated and apparently highly synchronized with stochastic events such as rainfall bouts, which may vary locally between study colonies^22^.

Why such rare genotypes persist in barn swallow populations is open to speculation. It is known that occasional spring cold spells, occurring after arrival of barn swallows to their breeding areas, can cause large mortality^25^,^26^. Clearly, the rare, delayed Q_7_/Q_8_ individuals may accrue a survival advantage under such intense natural selection episodes, and this may contribute to maintain *Clock* polymorphism in barn swallow populations. On the other hand, early arriving Q_6_/Q_7_ males may accrue a high fitness advantage by mating earlier if environmental conditions are favourable, even if the association between timing of spring arrival and reproduction was not as strong as in females in the study populations, since timing of reproduction predicts seasonal fitness in barn swallows^25^,^27^. Finally, we wish to point out that both here and in our previous studies we focused on the association between *Clock* gene polymorphism and phenology, but future studies will need to address whether the huge phenological variance observed among the most common Q_7_/Q_7_ genotype is mostly due to environmental effects or whether it can be explained by genetic variation at other phenological candidate genes.

To conclude, capitalizing on a unique dataset of individual year-round tracking data of a small long-distance migratory bird obtained by miniaturized light-level geolocators, the Swiss data are suggestive for a role of genetic polymorphism at a candidate phenological gene in shaping variation in migration traits. Despite the very low genetic variation and the consequently small sample of ‘rare’ genotypes, our findings foster the idea that *Clock* gene polymorphism may underlie phenological variation in avian species.

## Methods

### Study species, study sites and field procedures

The barn swallow is a small (ca. 20 g) Holarctic semi-colonial insectivorous passerine bird. European populations are largely migratory^28^. Birds move southwards in September-October, and winter in sub-Saharan Africa, where they undergo their annual complete plumage moult. During wintering, birds are mostly stationary^22^. They then leave for spring migration in February-March and reach their breeding sites mid-March to late May^22^. Females lay one to three clutches (3–7 eggs) per season^28^. In Europe, barn swallows mostly breed in small colonies settled in rural buildings (farms, cowsheds, stables)^29^.

The study was carried out at three study sites, one in southern Switzerland, in an Alpine valley floor (Magadino, 46°09’ N, 8°55’ E, 211 m a.s.l.) and two in the Po Plain (northern Italy; Lombardy, 45°19’ N, 9°40’ E, 60 m a.s.l.; Piedmont, 45°33’ N, 8°44’ E, 160 m a.s.l.) (details in Scandolara *et al*.^29^,^30^ and Liechti *et al*.^22^). We captured barn swallows at several colonies during two breeding seasons (2010 and 2011). A very large sample (640 individuals; 2010, n = 310; 2011, n = 330) of adult birds (i.e. at least 1 year old) was equipped with miniaturized light-level geolocators weighting on average 0.68–0.77 g (3.74–4.14% of body mass)^30^. During the subsequent breeding season we retrieved 124 geolocators (2010, n = 78; 2011, n = 46)^22^. We collected a small blood sample from birds that successfully returned to their breeding colony with the geolocator. Blood was collected from the brachial vein into heparinized capillary tubes, stored in a cool bag and subsequently kept at −20/−80 °C. Birds were marked with individual colour rings upon capture and were assigned to their nests by direct observation^17^,^30^. Laying date (Julian date of first egg laying) was determined for most individual by means of frequent inspections of nest content (details in Scandolara *et al*.^30^).

All procedures were performed in accordance with the Swiss and Italian regulations concerning scientific investigations on bird species in the wild, and approved by the Office fédéral de l’environnement (OFEV, Division Espèces, écosystèmes, paysages; Switzerland) (permit n. F044-0799), by Regione Lombardia (auth. n. 329 issued on January 21, 2009 and n. 2141 issued on March 9, 2011) and by the Provincia di Novara (auth. n. 905 issued on March 21, 2011).

### Geolocator data analysis

Deriving accurate geographical positions from light-level data obtained from geolocators constitutes a major methodological challenge^31^. The full details and rationale of the analyses of barn swallow geolocator data are reported in Liechti *et al*.^22^. Hereafter we briefly summarize the interpretation of the phenological traits of interest for defining individual variation in the timing of the annual routine. First, based on standardized analyses of daily changes in sunrise and sunset time derived from light-level data (latitudinal estimates were not calculated during migration because the barn swallow migration periods coincide with the period around the Equinoxes, when data derived from geolocators do not allow to accurately calculate latitudes^22^,^31^), we assigned each daily position to a stationary or movement (migration) period. We then calculated the following phenological traits at the individual level: departure from the breeding colony (day of departure from the breeding colony, determined by means of visual inspection of variation in daily light-level profiles); arrival to the wintering area (day of the first stationary period south of the Sahara, i.e. south of 23.5°N); departure from the wintering area (day of the last stationary period south of the Sahara); arrival to the breeding colony (date of arrival to the breeding colony, determined by means of visual inspection of variation in daily light-level profiles). Among the 124 individuals that returned with the geolocator (see above), we could obtain complete phenological information (i.e. we could obtain phenological data until spring arrival date to the breeding colony) for 68 individuals only (2010, n = 37; 2011, n = 31), due to total or partial failure of the geolocators (e.g. battery failure), which did not allow recovering the entire migration path (see Table 1 in Liechti *et al*.^22^). For each of these complete tracks we determined the stationary wintering position (SWP) as the centre of density (mode) of longitude and latitude of all the daily positions, taking into account the stationary periods south of the Sahara^22^.

### Genetic analyses

Genomic DNA extraction was performed by alkaline lysis using 6 μl of blood in 100 μl of a 50 mM NaOH at 100 °C for 20 minutes. DNA was quantified using a spectrophotometer and diluted to a final concentration of 50–100 ng/μl. PCR amplification was performed using primers designed on the barn swallow genomic sequence: forward primer (5′-labelled with 6-FAM dye) [6FAM]GGGACAGGTGGTGACAGCTTATC and reverse primer CTGCTGATGGTCCTGCTGACT (Sigma-Aldrich)^17^; PCR fragments were screened for length polymorphism at *Clk*polyQcds by resolution and detection on a conventional DNA sequencing machine using a commercial fragment analysis service (Macrogen Inc., Seoul, Republic of Korea)^17^. The reliability of molecular data for the barn swallow *Clk*polyQcds locus has previously been confirmed by independently repeating the fragment analysis of several individuals^17^,^20^. Alleles were named according to the number of glutamine repeats predicted in the mature protein as Q_6_ to Q_8_, after sequencing the most common allele (Q_7_, 112 bp long, coding for a CLOCK protein containing a stretch of 7 glutamine repeats)^32^. We genotyped the vast majority (64 out of 68 individuals; 2010, n = 34; 2011, n = 30) of the birds for which we obtained complete migration tracks (missing genotypes were due to failed genotyping or missing samples).

### Statistical analyses

Since the genotype-phenology associations may differ between populations^18^, tests of associations between *Clock* polymorphism and phenology were conducted within population. First, we calculated the 95% non-parametric bootstrap CIs (*BCa* method, based on 5000 replicates; R-package *boot*, ver. 1.3–9^33^) of each phenological trait of Q_7_/Q_7_ individuals. We then checked whether the phenological values of the Q_6_/Q_7_ and Q_7_/Q_8_ individuals were included or not within the CIs. If a phenological value was outside the Q_7_/Q_7_ CI of a given trait, that individual was considered phenodeviant for that trait (i.e. it had a phenotype that significantly deviated from the phenotypic distribution of the reference genotype). Male and female data were pooled for analyses as there were no statistically significant differences between the sexes for most phenological traits, with the exception of departure from the breeding colony, that was 2 days later on average in males compared to females^22^. In addition, to completely remove between-year and between-sex differences^22^, we adopted a within-group centring procedure, subtracting from a given value the mean of its corresponding group (i.e. 2010 males, 2010 females, 2011 males, 2011 females). CIs were calculated for centred values as well. Data from the Lombardy and Piedmont study areas were pooled because both were in the same geographical area (Po Plain) and because of the small sample of rare genotypes at each study site (see Results and Discussion).

## Additional Information

**How to cite this article**: Bazzi, G. *et al*. *Clock* gene polymorphism and scheduling of migration: a geolocator study of the barn swallow *Hirundo rustica*. *Sci. Rep*. **5**, 12443; doi: 10.1038/srep12443 (2015).

## Supplementary Material

Supplementary Information
