# Supplementary material for: Clock gene polymorphism and scheduling of migration: a geolocator study of the barn swallow Hirundo rustica
Source: Sci Rep. 2015 Jul 22;5:12443. doi: 10.1038/srep12443 (PMC4510496; doi:10.1038/srep12443)

Supplementary Information

Clock gene polymorphism and scheduling of migration: a geolocator study of the barn swallow *Hirundo rustica*

Gaia Bazzi[1], Roberto Ambrosini[2], Manuela Caprioli[1], Alessandra Costanzo[1], Felix Liechti[3], Emanuele Gatti[1], Luca Gianfranceschi[1], Stefano Podofillini[1], Andrea Romano[1], Maria Romano[1], Chiara Scandolara[1,3], Nicola Saino[1] & Diego Rubolini[1]

1. Dipartimento di Bioscienze, Università degli Studi di Milano, via Celoria 26, I-20133 Milano, Italy

2. Dipartimento di Biotecnologie e Bioscienze, Università di Milano-Bicocca, Piazza della Scienza 2, I-20126 Milano, Italy

3. Swiss Ornithological Institute, Seerose 1, CH-6204 Sempach, Switzerland

Table S1. Descriptive statistics of phenological traits (day 1 = January 1st) for Q_7/Q_7 individuals from the Swiss and Po Plain population. Mean, sample size (round brackets), and non-parametric bootstrap 95% confidence interval (CI) (square brackets) are shown. Results were qualitatively similar if we controlled for differences among years in linear models with year and population as two-level fixed factors (see also Ref. 22), and if we include data for the Q_6/Q_7 and Q_7/Q_8 individuals (details not shown for brevity). In the Po Plain, we could not estimate departure from the breeding colony and arrival to the wintering area for two Q_7/Q_7 individuals.

**Phenological trait**	**Switzerland**	**Po Plain**
Departure from the breeding colony	249.3 (23)<br>[245.4; 251.8]	251.2 (33)<br>[248.8; 253.2]
Arrival to the wintering area	283.8 (23)<br>[280.8; 286.2]	281.5 (33)<br>[278.4; 285.3]
Departure from the wintering area	76.7 (23)<br>[72.3; 81.4]	71.5 (35)<br>[67.7; 75.9]
Arrival to the breeding colony	111.3 (23)<br>[107.0; 115.7]	101.1 (35)<br>[97.0; 104.6]

Table S2. Mean, sample size (round brackets), and non-parametric bootstrap 95% confidence interval (CI) (square brackets) of phenological traits for Q_7/Q_7 individuals from the Swiss and Po Plain population. Data for the Q_6/Q_7 individuals (mean value, with individual values in square brackets) and for the Swiss Q_7/Q_8 female are also shown. Data were centred within-groups to remove any between-year and between-sex variation (see Methods) and are expressed in days (difference from the mean). For the Swiss population, statistics of residuals from the linear regression between centred arrival date to the breeding colony values and centred latitude and longitude of the SWP (see Methods), thus removing the effects of individual variation in wintering positions on arrival date to the breeding colony (see Ref. 22), are also shown. In the Po Plain, we could not estimate departure from the breeding colony and arrival to the wintering area for two Q_7/Q_7 individuals.

**Phenological trait**	**Q_6/Q_7**	**Q_7/Q_7**	**Q_7/Q_8**
Switzerland			
Departure from the breeding colony	2.33<br>[2.33; 2.33]	-0.48 (23)<br>[-3.70; 1.74]	6.33
Arrival to the wintering area	-5.50<br>[-10.00; -1.00]	0.35 (23)<br>[-2.01; 2.67]	3.00
Departure from the wintering area	-11.50<br>[-16.00; -7.00]	0.10 (23)<br>[-5.01; 4.47]	20.67
Arrival to the breeding colony	-21.06<br>[-24.56; -17.56]	1.57 (23)<br>[-2.51; 5.79]	6.00
Arrival to the breeding colony (residuals)	-14.77<br>[-20.43; -9.10]	1.03 (23)<br>[-2.65; 4.61]	5.86
Po Plain			
Departure from the breeding colony	3.65<br>[1.80; 4.08; 5.08]	-0.33 (33)<br>[-2.53; 1.36]	-
Arrival to the wintering area	4.10<br>[-1.40; 6.85; 6.85]	-0.37 (33)<br>[-3.32; 3.61]	-
Departure from the wintering area	8.16<br>[-6.07; -1.40; 31.93]	-0.70 (35)<br>[-4.50; 3.54]	-
Arrival to the breeding colony	11.16<br>[0.00; 11.93; 17.93]	-0.85 (35)<br>[-4.92; 2.59]	-

Table S3. Mean, sample size (round brackets), and non-parametric bootstrap 95% confidence interval (CI) (square brackets) of phenological traits (day 1 = January 1st) for Q_7/Q_7 males and females from the Swiss population. Data for the two Q_6/Q_7 males are shown in the leftmost column (mean value, with the two individual values in square brackets), while data for the Q_7/Q_8 female are shown in the rightmost column.

Phenological trait	Q_6/Q_7	Q_7/Q_7 males	Q_7/Q_7 females	Q_7/Q_8
Departure from the breeding colony	257.0 [257; 257]	250.2 (14) [245.6; 253.0]	248.0 (9) [240.5; 252.4]	251
Arrival to the wintering area	273.5 [269; 278]	284.6 (14) [280.4; 287.4]	282.6 (9) [278.2; 286.2]	288
Departure from the wintering area	59.5 [55; 64]	74.6 (14) [68.4; 79.6]	80.1 (9) [73.2; 88.6]	113
Arrival to the breeding colony	79.5 [76; 83]	110.3 (14) [105.4; 115.3]	112.8 (9) [104.8; 120.9]	130

Figure S1. Frequency distribution of phenotypic values of the Q_7/Q_7 individuals and phenology of the Q_6/Q_7 individuals in the Po Plain. a) Departure from the breeding colony; b) arrival to the wintering area; c) departure from the wintering area; d) arrival to the breeding colony. The horizontal boxes above histograms shows the mean and 95% non-parametric confidence interval (CI) of the Q_7/Q_7 phenotypic distribution, while the horizontal lines show the standard deviation. Dots show the phenotypic values of the Q_6/Q_7 individuals. The x- and y-axis are on the same scale as those of the corresponding panels of Fig. 1, to facilitate comparisons of phenology between populations.

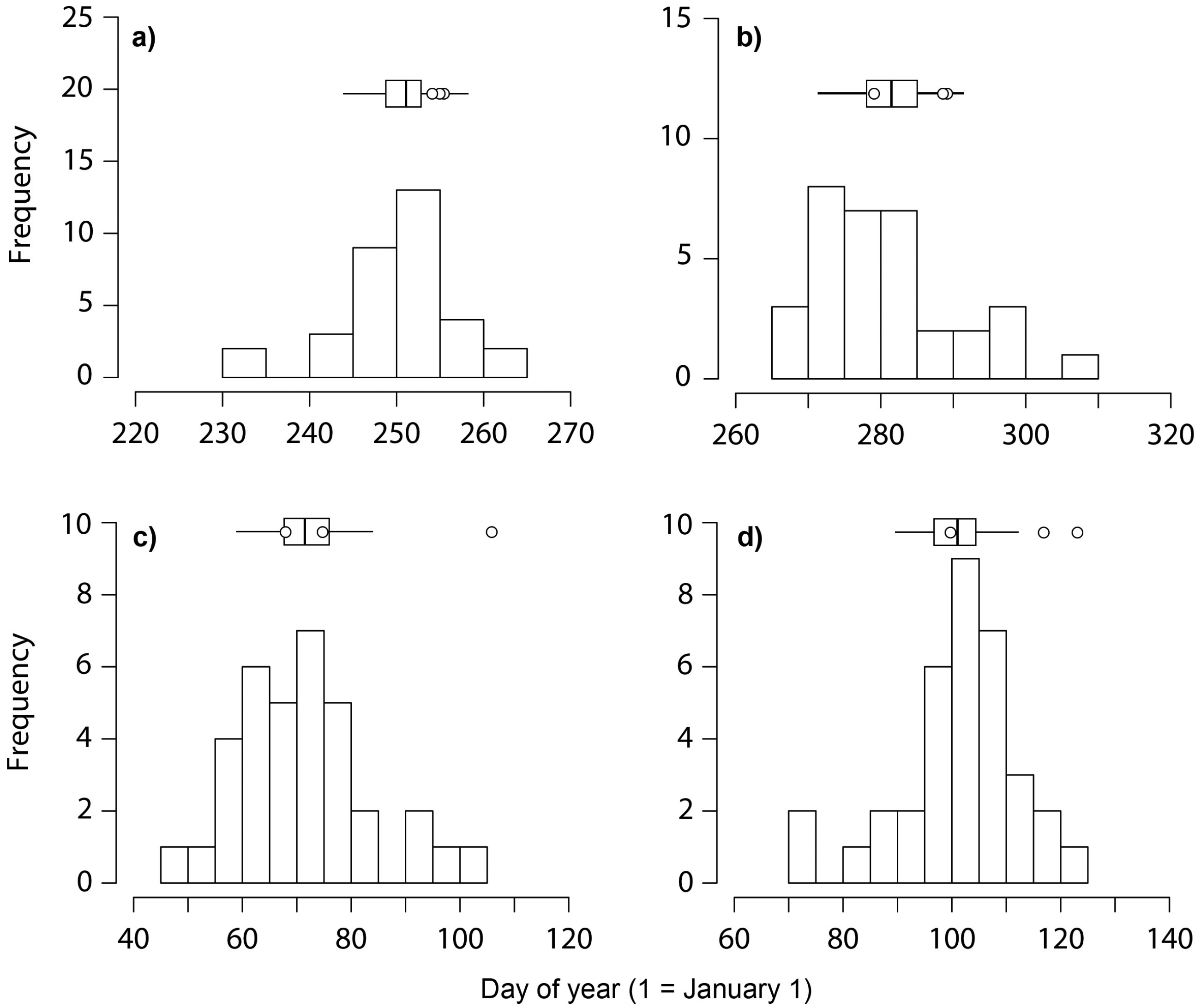

Supplement: Supplementary Information [file srep12443-s1.pdf]
